# Mechanisms and Functions of ATP-Dependent Chromatin-Remodeling Enzymes

**DOI:** 10.1016/j.cell.2013.07.011

**Published:** 2013-08-01

**Authors:** Geeta J. Narlikar, Ramasubramanian Sundaramoorthy, Tom Owen-Hughes

**Affiliations:** 1 Biochemistry and Biophysics, Genentech Hall 600, 16th Street, University of California, San Francisco, San Francisco, CA 94158, USA; 2 Centre for Gene Regulation and Expression, College of Life Sciences, University of Dundee, Dundee DD1 5EH, UK

## Abstract

Chromatin provides both a means to accommodate a large amount of genetic material in a small space and a means to package the same genetic material in different chromatin states. Transitions between chromatin states are enabled by chromatin-remodeling ATPases, which catalyze a diverse range of structural transformations. Biochemical evidence over the last two decades suggests that chromatin-remodeling activities may have emerged by adaptation of ancient DNA translocases to respond to specific features of chromatin. Here, we discuss such evidence and also relate mechanistic insights to our understanding of how chromatin-remodeling enzymes enable different in vivo processes.

## Main Text

### Introduction

The packaging of eukaryotic DNA into chromatin provides a means to partition the genome into transcriptionally active and transcriptionally repressed regions. Different patterns of partitioning allow diverse transcriptional programs to arise from a single genetic blueprint. The establishment of specific chromatin states during the course of development as well as their maintenance through the disruptive events of transcription, DNA replication, and DNA repair require rapid rearrangements of chromatin structure. ATP-dependent chromatin-remodeling enzymes provide a means of generating such changes in chromatin structure.

These enzymes are often referred to as Snf2- or SWI/SNF-related enzymes. This stems from the characterization of the yeast SWI/SNF complex as the first ATP-dependent chromatin-remodeling enzyme (Côté et al., 1994). The Snf2 protein contains a Walker box, which is one element of a series of seven amino acid sequence motifs that are conserved between the Snf2 protein and the superfamily 2 (SF2) grouping of helicase-related proteins (Gorbalenya and Koonin, 1993). Proteins related to Snf2 have since been identified within the genomes of all eukaryotes. Based upon sequence homology within the ATPase core, these can be assigned into some 24 distinct subfamilies (Flaus et al., 2006). Many of these subfamilies are broadly conserved through evolution (Table 1).

Together, the different subfamilies of chromatin-remodeling enzymes catalyze a broad range of chromatin transformations that includes sliding the histone octamer across the DNA, changing the conformation of nucleosomal DNA, and changing the composition of the histone octamer. These biochemical activities are remarkable given the underlying mechanistic challenges. The substrate, a nucleosome, is structurally complex and contains DNA tightly bound to the histone octamer. Somehow, chromatin-remodeling enzymes have to disrupt DNA-histone interactions while contending with and leveraging the structural constraints placed by the histone octamer. Here, we largely focus on recent observations relating to the ISWI and Chd1 proteins and the implications for the mechanisms via which these enzymes act. We then relate their different biochemical outputs to their emerging biological roles.

### A Core DNA Translocase Function

Interesting insights into mechanism have been derived from observations that many members of the SF2 family share the ability to translocate along nucleic acids in an ATP-dependent manner (Singleton et al., 2007), raising the possibility that the remodeling complexes also have DNA translocase activity. This is indeed the case, and single-molecule approaches have been used to detect translocation by SWI/SNF and RSC complexes directly as a result of the ability of these complexes to generate ATP-dependent loops in DNA molecules (Lia et al., 2006; Saha et al., 2002; Zhang et al., 2006). For RSC, the directionality of translocation appears to be 3′→5′ (Saha et al., 2002). However, several studies have detected that a proportion of translocation events result in an ATP-dependent reversal of loop formation (Lia et al., 2006; Sirinakis et al., 2011). This may reflect a capacity of the ATPase lobes to switch from engagement with one strand to the other at low frequency.

The processivity of translocation events on naked DNA has been most sensitively measured using a tethered subcomplex of RSC components. These translocation events occur at a speed of 25 bp per second with a mean processivity of 35 bp and are likely to be made up of small steps of the order of 2 bp, which can result in the generation of forces of up to 30 pN (Sirinakis et al., 2011). More recently, repositioning of nucleosomes by ISWI complexes has been shown to occur in steps of 1 bp (Blosser et al., 2009; Deindl et al., 2013). This makes it likely that Snf2-related enzymes like other SF2 translocases share an elementary step size of 1 bp per ATP molecule hydrolyzed (Singleton et al., 2007).

An enzyme that translocates along the helical axis of DNA with a step size of 1–3 bp is expected to generate torsion in DNA, and the accumulation of such superhelical torsion has been detected (Lia et al., 2006). However, the rate at which torsion is generated is less than that anticipated to result from tracking the DNA backbone, suggesting that some torsion is lost via a slippage mechanism. The combined effect of translocation and torsion could generate incremental distortions in DNA-histone contacts that are presumably harnessed by different enzymes to achieve different outcomes. To understand how these changes can be used to reconfigure nucleosomes, it is important to consider where the tranlocase lobes engage with nucleosomal DNA.

**Figure 1:**
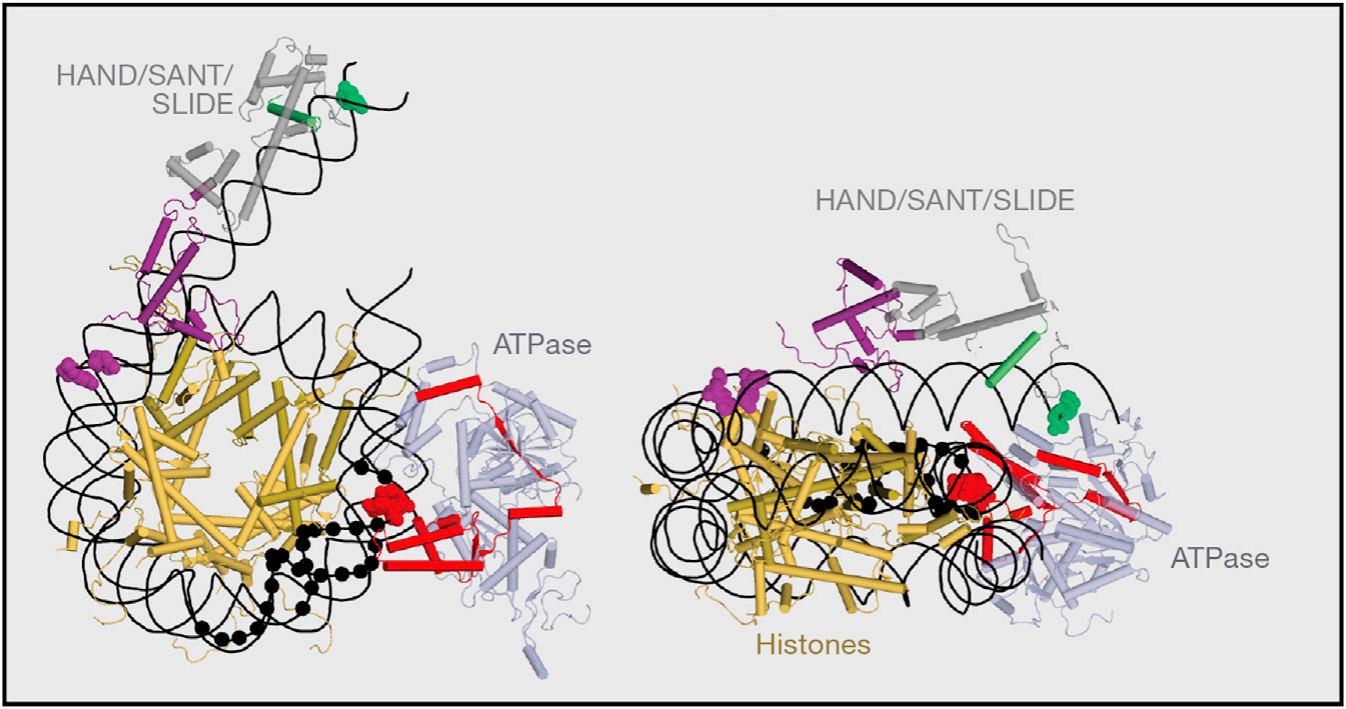
Model of Isw2 Nucleosome Interactions The Isw2 complex represents a paradigm for nucleosome remodeler interactions, as the sites at which peptides derived from translocase lobe 1 and the HAND/SANT and SLIDE domains interact with nucleosomal DNA (black) have been determined by directed crosslinking (Dang and Bartholomew, 2007). The HAND/SANT SLIDE domain of Isw2 (gray) is modeled using the equivalent region of the Isw1 (Yamada et al., 2011). Two peptides from this region of the ISW2 complex (green and purple) crosslink to the bases shown in the space-fill of the same color. The RecA lobes (light-blue) are modeled using the structure of zebrafish Rad54 (Thomä et al., 2005) and the peptide shown in red crosslinked to the bases shown in red space-fill. The precise details of how each domain is docked are not known, as the specific amino acids that form crosslinks within each peptide are not known. Black dots indicate the positions of single-nucleotide gaps that interfere with nucleosome sliding (Zofall et al., 2006). Core histones are shown as yellow.

Experiments involving site-directed crosslinking and DNA gaps and nicks have together suggested that the translocase lobes of ISWI and SWI/SNF enzymes engage nucleosomal DNA at an internal location, ∼20 bp away from the dyad (super helical location [SHL] −2/+2). A crosslinker attached to bases 17 and 18 bp from the dyad axis interacts with a 128 amino acid peptide comprised of the N-terminal translocase lobe of the Isw2 protein within the context of an ISW2-nucleosome complex (Dang and Bartholomew, 2007). The length of the crosslinker (∼10 Å) and the approximation of the position of the crosslinked amino acid within each peptide do not allow for identifying the precise location of the ATPase lobes (Figure 1). Yet the data provide important mechanistic constraints by locating the ATPase lobes in the approximate vicinity of SHL–2/+2. Gaps and nicks that cover 1–5 bp over the SHL–2/+2 region of nucleosomal DNA inhibit nucleosome movement by ISWI complexes as well as SWI/SNF and RSC complexes, whereas similar gaps and nicks at other locations do not have large inhibitory effects (Saha et al., 2005; Schwanbeck et al., 2004; Zofall et al., 2006). These results suggest that the translocase domains of remodeling enzymes act on DNA located at an internal region of the nucleosome. However, the 3′-5′ preference observed on naked DNA has not been consistently observed on nucleosomal DNA. This raises the possibility that the gaps affect the generation of an altered DNA structure instead of only affecting DNA translocation.

**Figure 2:**
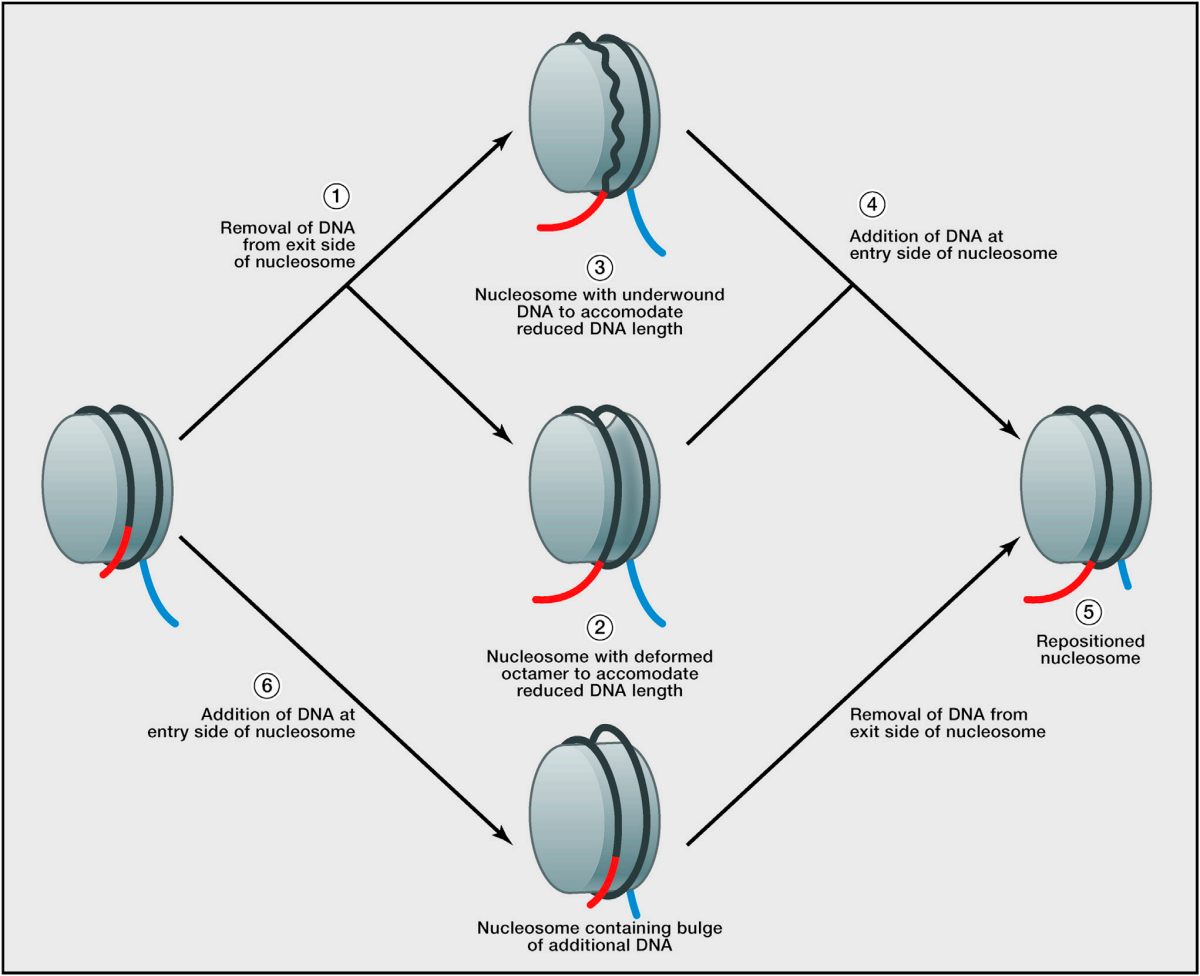
Mechanisms for Nucleosome Repositioning (1) Single-molecule measurements indicate that DNA is first removed from nucleosomes on the exit side (red) (Deindl et al., 2013). As a result, the intermediates in repositioning contain a deficit of DNA that has been measured as between 4 and 7 bp. This could be accommodated as a change in the conformation of the octamer (2), a reduction in DNA twist (3), or a combination of these. DNA is subsequently drawn into the nucleosome on the destination side (blue) (4), allowing the nucleosome to return to a more normal conformation 3 bp further along the DNA (5). This contrasts with previous models in which DNA was proposed to be drawn into the nucleosome prior to being removed (6). Note that, although DNA appears to enter and leave the nucleosome in 3 bp steps, these are likely to arise from three successive 1 bp movements of the remodeling enzyme.

Using an elegant single-molecule approach, Deindl et al. have directly tracked the motion of DNA across the surface of the histone octamer during the course of repositioning directed by the ISW2 complex (Deindl et al., 2013). This study showed that, although the translocase tracks along DNA, pushing DNA out of the nucleosome in 1 bp increments, DNA is drawn into the nucleosome from the other side units of 3 bp. Counterintuitively, DNA exits the nucleosome core before it enters in from the other end. Remarkably, in the initial repositioning reaction, 7 bp of DNA are removed from a nucleosome in 1 bp increments before any DNA is drawn into the nucleosome from the other side. The removal of 7 bp is speculated to be a prerequisite for generating strain required to draw DNA in from the opposite side of the nucleosome. Once this strain has been generated, DNA is pulled into the nucleosome in successive increments of 3 bp. As a result, there is a deficit of between 4 and 7 bp during the course of repositioning. This is, in effect, opposite to previously proposed models involving extra DNA being drawn into the nucleosome in the form of loops before it could exit from the other side (Figure 2) (Clapier and Cairns, 2009).

How could such deficits in DNA content be accommodated within the nucleosome? A clue may come in the form of nucleosomes crystallized on different DNA sequences. These show that a deficit can be accommodated through underwinding of DNA and that this is favored at specific locations on the octamer, SHL2 and SHL5 (Tan and Davey, 2011). The transit of DNA through these locations may represent an important step during ATP-dependent remodeling, perhaps reflected by the sensitivity to introduction of nicks and gaps at these locations. Although crystal structures illustrate a means to reduce the DNA content of a nucleosome by 1 bp, it is not immediately obvious how it can be reduced by 4 or 7 bp. An intriguing possibility is that each superhelical location can accommodate a 1 bp reduction in DNA content, and once this occurs at each of the seven superhelical locations present in one half of the nucleosome, then additional DNA is dragged into the nucleosome. However, it is not clear that a nucleosome could withstand such extreme unwinding, in which case a more substantial change in the conformation of the histone octamer will be required.

To date, there is no direct evidence for conformational changes within the histone octamer during ATP-dependent remodeling. However, recent data on nucleosome dynamics suggest that nucleosomes are capable of adopting alternative conformations in which the interface between H2A-H2B dimers and the H3-H4 tetramer is altered (Böhm et al., 2011). If analogous conformational changes can occur during remodeling, this would significantly increase the types of DNA distortions that are possible within a nucleosome. In light of this possibility, it makes sense to consider that the histone octamer is not simply a monolithic roadblock for a translocating remodeling enzyme but, rather, plays a more active role in the remodeling process. Indeed, it has long been known that specific histone modifications, such as acetylation, act as recruitment devices and that specific histone tails, such as the H4 tail, act as catalytic regulators (reviewed in Clapier and Cairns, 2009).

The observations described above are consistent with the possibility that chromatin-remodeling activities evolved from a primitive DNA-translocating core (Fairman-Williams et al., 2010). The biophysical properties of this core—that it moves in 1 bp steps and can generate high forces yet is not highly processive—make it well suited for manipulation of DNA-protein contacts. This has utility in regulating nonnucleosomal as well as chromatin-related complexes, as described further in the sections below. Furthermore, activities such as those of ISWI complexes, which position a nucleosome at the midpoint between its neighbors, require the chromatin remodelers to be more than just a snowplow that tracks along DNA, displacing any histones that are encountered. How might chromatin remodelers achieve the sophisticated gymnastics and regulation that seem necessary to achieve such well-defined outcomes? Part of the answer may lie in how the accessory domains regulate the activity of the translocase core, which we discuss in the next section.

### Regulating the Translocase

**Figure 3:**
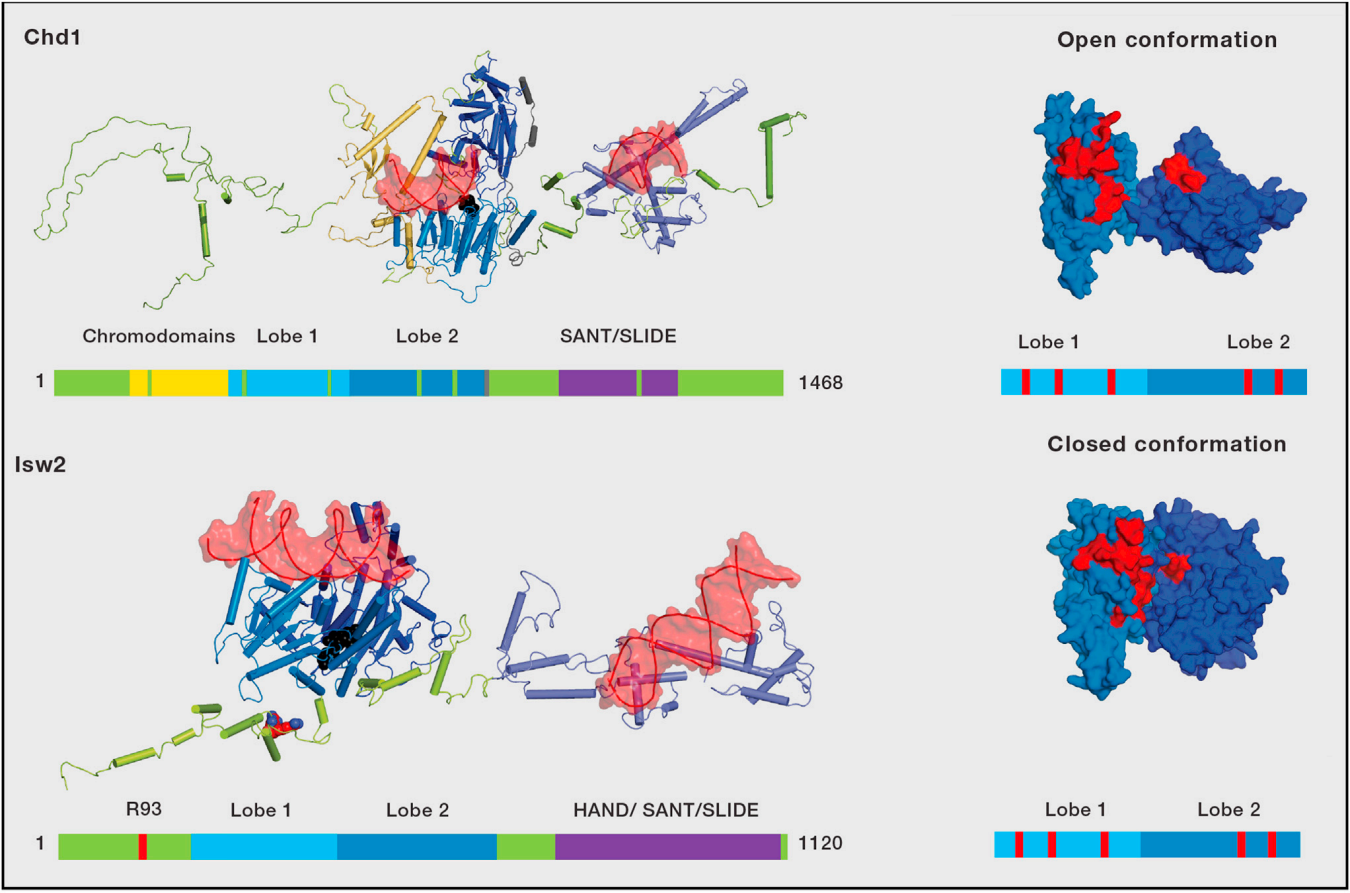
Structural Models for Chd1 and Isw2 The chromodomains, translocase lobes (Hauk et al., 2010), and SANT/SLIDE DNA-binding domain (Sharma et al., 2011) of Chd1 are colored yellow, blue, and purple, as indicated in the schematic. The structure of linker sequences was crudely modeled based upon secondary structure prediction to indicate their scale rather than conformation. To the right, the helicase lobes are shown as space-fill, with the conserved DNA-binding motifs I, II, and III of lobe I and motifs V and VII of lobe II indicated in red. These conserved motifs are observed to be held in an open conformation that is likely to be inefficient for ATP-dependent DNA translocation. A similar model is shown for Isw2. In this, the HAND-SANT-SLIDE domain is modeled on Isw1 (Yamada et al., 2011), and the ATPase lobes are modeled using the structure of Zebrafish Rad54 in a configuration close to the closed conformation likely to be active for DNA translocation (Thomä et al., 2005). For both Chd1 and Isw2, accessory sequences contribute to the regulation of catalytic activity, and this may well involve changes in the alignment of the ATPase lobes. For example, the chromodomains of Chd1 and R93 of the ISWI protein (in red space-fill) confer negative autoregulation (Clapier and Cairns, 2012; Hauk and Bowman, 2011). This region also undergoes a conformational change upon DNA binding (Mueller-Planitz et al., 2013). In contrast the SANT-SLIDE domains of both proteins confer positive regulation (Hota et al., 2013; McKnight et al., 2011). DNA fragments bound to the SANT/SLIDE domains and modeled into the translocase domain are shown in red space-fill. However, it should be noted that, as the conformation of linker sequences (green) is not known, it is not possible to infer the orientation of the two bound DNA fragments.

The translocating core of SF2 proteins consists of 2 RecA-like domains or lobes (Singleton et al., 2007). Conserved sequences from each domain are brought together in a closed conformation to form surfaces capable of interacting with and hydrolyzing ATP and for binding nucleic acids (reviewed in Hauk and Bowman, 2011). Coupled with ATP binding and hydrolysis, reconfiguration of the interface between the RecA-like domains is thought to drive translocation along DNA or RNA. As a result, the conformation of the RecA-like domains with respect to each other is anticipated to be somewhat dynamic. Consistent with this, the structures of the RecA-like domains from Snf2-related enzymes have been observed in different conformations. For example, the lobes of the Sulpholobus Snf2 homolog SSO1653 are flipped 180° with respect to the closed conformation (Dürr et al., 2005). In the case of the yeast Chd1 protein, the motifs that are critical for ATP hydrolysis are held apart in an open conformation that is unlikely to be active (Hauk et al., 2010) (Figure 3). In contrast, in the case of Zebrafish Rad54, the helical lobes are close to the closed and active conformation (Thomä et al., 2005). This raises the question of the functional significance of the different conformations adopted by the RecA domains.

An attractive concept is that the adoption of an inactive conformation provides a means of regulation. There are several reasons why it may be important that the activity of Snf2-related enzymes is regulated. First, as even simple eukaryotes such as budding yeast encode some 17 Snf2-related proteins, their combined abundance could represent a significant burden on cellular ATP levels if constitutively active. Second, though Snf2-related enzymes share related translocase domains, these have specificity for different substrates such as TBP in the case of Mot1 and different types of nucleosomes in the case of other remodeling enzymes. Third, as we mentioned in the previous section, few if any Snf2-related proteins are likely to act as molecular snowplows simply tracking along DNA indefinitely and are likely to require sophisticated regulation by the local context.

Regulation in many cases is likely to be specific for enzymes performing related tasks. This specificity is likely to be in part conferred by the translocating core, as sequence alignments of this region alone are sufficient to distinguish different subfamilies. However, as accessory domains are also conserved between subfamilies, it is likely that these also contribute to both regulation and specificity. Recent observations with ISWI and Chd1 remodelers provide evidence that indicates mechanistic roles for the different conformations of the RecA domains and for the accessory domains in regulating these different conformations. These examples are reviewed below.

#### Positive Regulation of Binding and Catalysis

One of the best-characterized accessory domains are the HAND-SANT-SLIDE domains located C terminal to the ATPase domains within the *Drosophila* ISWI protein (Grüne et al., 2003). Crosslinking approaches have localized the binding of the SANT and SLIDE domains of ISWI complexes to linker DNA (Dang and Bartholomew, 2007) (Figure 1). Subsequently, a structurally related SANT-SLIDE domain has been identified in an equivalent position within the yeast Chd1 protein (Ryan et al., 2011). The SANT domain is related to the myb DNA-binding domain, and the isolated domains bind DNA independently (Sharma et al., 2011; Yamada et al., 2011) (Figure 3).

Chd1 proteins from which the SANT-SLIDE domains have been ablated by mutation or deletion have considerably lower affinity for DNA or nucleosomes (Grüne et al., 2003; Ryan et al., 2011). Orders of magnitude of higher concentrations of ISWI and Chd1 proteins deleted for their SANT-SLIDE domains are required to observe nucleosome sliding at levels equivalent to full-length proteins. This is consistent with a role for the SANT-SLIDE domain in targeting the remodeling enzyme to nucleosomal DNA.

In the context of the isolated ISWI protein, deleting the HAND-SANT-SLIDE domain modestly reduces the maximal rates of nucleosome remodeling (Mueller-Planitz et al., 2013). However, within a native ISW2, complex mutation of just the SLIDE domain results in only small defects in the affinities for DNA or nucleosomes (Hota et al., 2013). Much larger defects in the rates of ATP hydrolysis and nucleosome remodeling are observed (Hota et al., 2013). In the case of Chd1 as well, the directionality of nucleosome movement changes upon deleting the SANT-SLIDE domain (McKnight et al., 2011). The intact Chd1 protein has a tendency to reposition nucleosomes to locations close to the center of short DNA fragments (Stockdale et al., 2006), consistent with its native function spacing arrays of nucleosomes (Lusser et al., 2005). Deletion of the SANT-SLIDE domains results in an enzyme with residual sliding activity that now tends to reposition nucleosomes toward the ends of short DNA fragments (McKnight et al., 2011). This may be the default outcome when a nonregulated translocase encounters a nucleosome (Finkelstein et al., 2010). Remarkably, fusion of an exogenous DNA-binding domain restores robust sliding activity in the absence of the endogenous SANT-SLIDE domain (McKnight et al., 2011; Patel et al., 2012). The directionality of sliding in these chimeric Chd1 proteins is such that nucleosomes are repositioned toward the DNA-binding site for the heterologous DNA-binding domain. These results imply that the linker DNA-binding domains also play important mechanistic roles subsequent to nucleosome binding.

How might the SANT-SLIDE domain regulate the directionality and rate of nucleosome mobilization? As mentioned above, the SANT-SLIDE domain of the ISW2 protein crosslinks with linker DNA, and this extends up to 30 bp from the edge of a nucleosome (Dang and Bartholomew, 2007) (Figure 1). It is possible that a similar region is bound by the SANT-SLIDE domain of the Chd1 protein. In both cases, the SANT-SLIDE domain may help to bind and guide the movement of the linker DNA into the nucleosome in a manner that is coordinated with the actions of the translocase domain. Consistent with this, both ISWI- and Chd1-remodeling enzymes have been observed to show increased activity for nucleosomal substrates with extended linker DNA (Kagalwala et al., 2004; Stockdale et al., 2006; Whitehouse et al., 2003; Yang et al., 2006). This increase in activity appears to be attributable to both more efficient DNA binding and a faster rate of translocation when these spacing enzymes are able to engage linker DNA. Recent data further show that mutating the SLIDE domain within the ISW2 complex reduces the ability of the complex to move the longer linker DNA into the nucleosome (Hota et al., 2013).

A consequence of moving linker DNA into a nucleosome is that the length of the linker will decrease progressively. A negative-feedback loop is formed in which the shortening of the linker DNA slows down further movement of the nucleosome. At some point, the linker on the other side of the nucleosome is likely to be a better substrate for repositioning, resulting in the movement of the nucleosome in the opposite direction. This process provides a means by which a chromatin-remodeling enzyme can relocate a nucleosome to a position close to the midpoint between its neighbors via a process of continuous sampling. Although this process has been proposed for some time (Kagalwala et al., 2004; Yang et al., 2006), a major step toward confirming such a mechanism results from the direct observation of single nucleosomes under the action of a remodeling enzyme. Blosser et al. observed that, at steady state, nucleosomes continuously bound by the ISWI-containing ACF complex move back and forth (Blosser et al., 2009). In principle, such back and forth motion could result from the dissociation of an ACF complex from one side of a nucleosome and its association with the linker DNA on the other side. However, Blosser et al. performed three color experiments that enabled bidirectional motion to be observed within a single binding event (Blosser et al., 2009). At the same time, Racki et al. showed that nucleosomes are repositioned more efficiently when two ACF complexes engage with a single nucleosome (Racki et al., 2009). These results suggested a model for ACF in which each of the two ACF protomers takes a turn moving the nucleosome in one direction, and the complex with access to the longest linker DNA more often moves the nucleosome.

#### Negative Regulation of Binding and Catalysis

In addition to the SANT-SLIDE domains, there are additional conserved sequences present in both the N- and C-terminal regions of ISWI and Chd1 proteins. A breakthrough in this respect arose from the crystallization of the combined chromodomain and translocase-related domains of Chd1 (Hauk et al., 2010). The most striking feature of the structure is that the chromodomains are located in the cleft between the two ATPase lobes in a location that interferes with their alignment in the closed configuration required for ATP hydrolysis and occludes the residues that are likely to interact with DNA (Figure 2). This led the authors to propose that Chd1 is subject to negative regulation via the chromodomains. Supporting this, a Chd1 protein in which the chromodomains have been deleted hydrolyzes ATP faster than the intact protein. Point mutations disrupting the interface between the chromodomains and translocase domains as well as deletion of the chromodomains increase DNA binding and DNA-stimulated ATP hydrolysis, consistent with the chromodomains competing with DNA for access to the translocase lobes. In addition, deleting the chromodomains partially relieves the dependence on the histone H4 tail, which is an important nucleosomal epitope required for maximal remodeling by Chd1 proteins. Interestingly, however, mutating the chromodomains has a negative effect on the rate of nucleosome sliding. Thus, in addition to playing a role in regulating ATP hydrolysis, chromodomains appear to help couple energy derived from ATP hydrolysis to nucleosome repositioning.

This form of negative allosteric regulation is unlikely to be unique to chromodomains. The ISWI protein does not contain chromodomains, but point mutations of two arginine residues adjacent to an acidic patch N terminal to the translocase domains have been found to increase ATPase activity (Clapier and Cairns, 2012). Upon binding to DNA, a change in proteolytic cleavage has recently been reported to occur at precisely this site (Mueller-Planitz et al., 2013). ISWI proteins also use the histone H4 tail for mobilizing nucleosomes but to a greater extent than Chd1. Clapier and Cairns therefore propose that recognition of the H4 tail by the active site relieves the autoinhibition caused by the region containing the two arginines. Interestingly, although mutating the two arginines increases overall remodeling rates, the rates remain sensitive to the presence of the H4 tail basic patch. This result is analogous to the results with Chd1 described above and suggests that, in both cases, the H4 tail plays a catalytic role in addition to helping to displace an autoinhibitory module.

Together, the data imply that the basic residues in ISWI and the chromodomains of Chd1 negatively regulate ATPase activity, and upon recognition of specific nucleosomal features, this negative regulation is removed and translocation can proceed unimpeded. That this loss of negative regulation involves a realignment of the translocase lobes into the closed conformation has yet to be directly shown. However, it is notable that the ATPase domains of the Sulfolobus Snf2-related protein SSO1653 have been observed to move closer together upon binding to DNA (Lewis et al., 2008).

#### Integrating Information from Different Substrate Cues

These observations illustrate how the non-ATPase domains can influence the activity of the translocating core either positively or negatively and suggest models for how specific features of a nucleosome, such as linker DNA, and the H4 tail can be used to gate the effects of these domains. The information provided by these substrate cues appears to have three types of effects: increasing recruitment, increasing rates of ATP hydrolysis and remodeling, and increasing the efficiency with which ATP hydrolysis is coupled to remodeling.

Remodeling enzymes thus may have gradually evolved from primitive nucleic acid translocases by co-opting different features of a nucleosome for the purpose of regulating the basic movements of the RecA lobes. This process of combinatorial recognition provides an opportunity for kinetic proofreading mechanisms to discriminate between the correct and the incorrect substrates (Blossey and Schiessel, 2008; Narlikar, 2010). For example, flanking DNA could be “read” twice—once by its ability to stimulate ATP hydrolysis and then by its ability to stabilize an activated intermediate that is generated upon ATP hydrolysis. Such additional mechanisms of specificity beyond effects on binding and catalysis may be important in vivo to minimize the remodeling of chromatin templates that do not, for example, have the correct modification status.

Although the discussion above mainly focuses on linker DNA and the histone H4 tail, there is a large range of nucleosomal and nonnucleosomal epitopes that can be recognized by accessory domains and additional subunits present in remodeling complexes (Table S1 available online). The additional nucleosomal epitopes that have been characterized to date include specific histone marks (described in Clapier and Cairns, 2009) and the nonnucleosomal epitopes include transcription factors and branched DNA structures (Table S1). These provide a means of adapting the action of the motor domains to a diverse range of functions. This occurs, in part, as a result of accessory domains providing a means of targeting recruitment of enzymes to specific genomic features. However, there is no reason why a subset of these epitopes should not have effects on catalysis in addition to or instead of recruitment, as observed with the H4 tail and flanking DNA.

Accessory domains and subunits are often arranged in large complexes and have the potential to substantially affect the outcome of remodeling by regulating the location and activity of the ATPase domain. For example, in the case of the ISWI protein, accessory subunits such as Acf1 can alter the directionality of repositioning and increase the efficiency of generating evenly spaced chromatin (Eberharter et al., 2001; Fyodorov et al., 2004; He et al., 2006). What is much needed to provide further insight into the mechanistic roles of the different accessory domains is a better understanding of how the domains are organized with respect to each other. The cases that have been studied to date indicate compact organization with close proximity between accessory and ATPase domains (Hauk et al., 2010; Morra et al., 2012; Watson et al., 2012; Bétous et al., 2012), meaning that they are well placed to influence the ATP hydrolysis cycle in response to specific nucleosomal and nonnucleosomal epitopes. As a result, the diversity range of accessory domains and subunits provides a means of adapting the action of the ATPase core to diverse biological functions such as DNA repair, recombination, and replication in addition to transcription.

### Emerging Roles for ATP-Dependent Remodeling Enzymes in Chromatin Organization In Vivo

**Figure 4:**
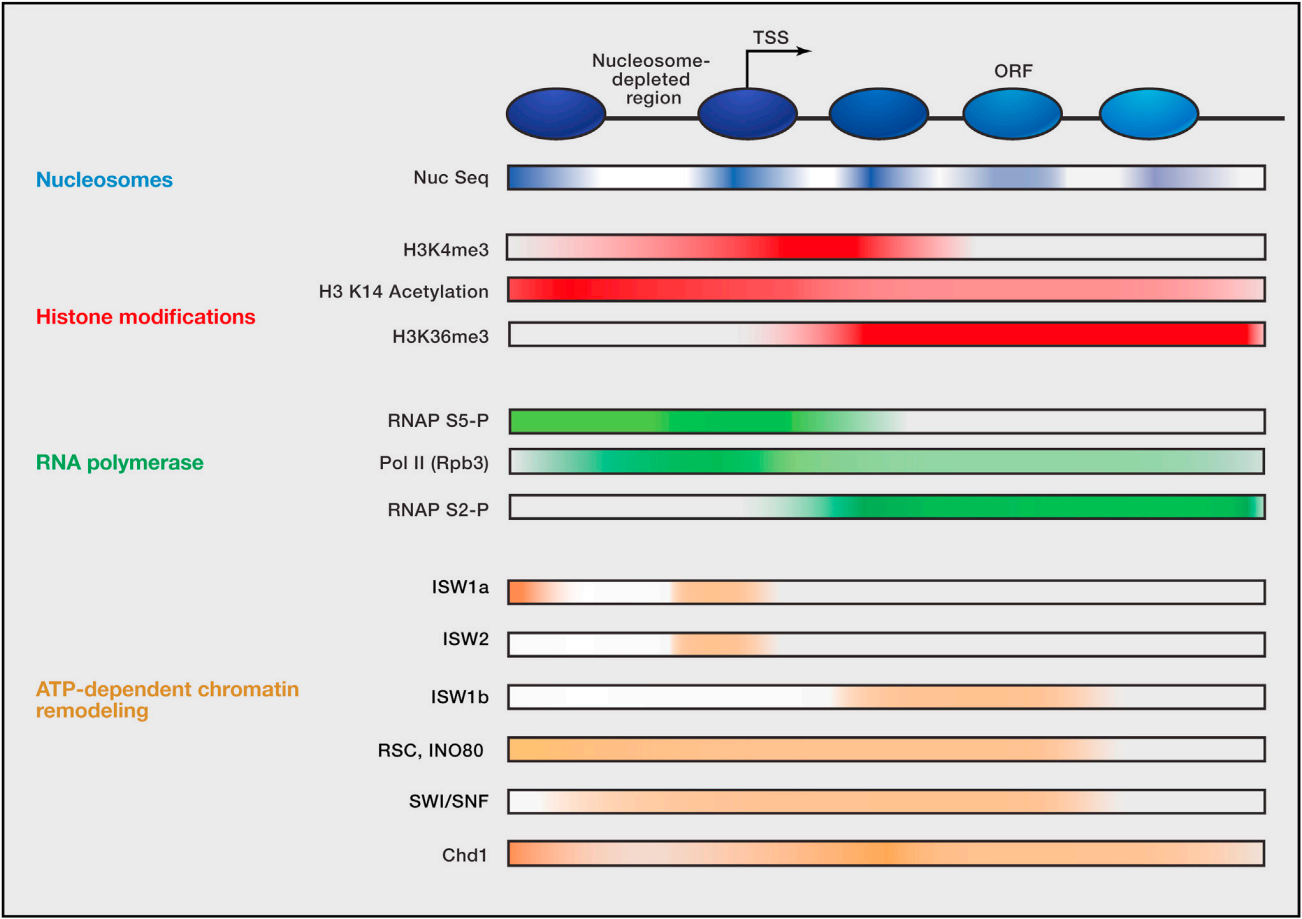
Organization of Chromatin-Remodeling Enzymes with Respect to Transcribed Genes A schematic representation organization of chromatin-related factors with reference to the transcriptional start site (TSS). The genome-wide distributions of nucleosomes and posttranslational modifications to histones and RNA polymerase subunits reveal that many of these factors are organized with respect to transcribed genes (reviewed by Rando and Chang, 2009). More recently, it has become apparent that ATP-dependent remodeling enzymes also show distinct distributions with respect to transcribed genes where they influence nucleosome organization (Gkikopoulos et al., 2011; Yen et al., 2012). Furthermore, in some cases, the action of remodeling enzymes can influence the distribution of histone modifications and variants, whereas in other cases, modifications instruct the action of remodelers. This interplay between histone modifications and ATP-dependent chromatin modifications acts to sculpt the chromatin landscape on a genome scale and is likely to involve further integration with transcriptional elongation factors and additional factors acting to regulate chromatin organization, such as histone chaperones.

Chromatin-remodeling enzymes appear to use the ability to translocate on DNA and the ability to respond to nucleosomal features to achieve a diverse range of biochemical outputs. These diverse outputs, in turn, appear to be linked to specialization of biological function. The rapid development of high-resolution genomic approaches provides new opportunities to relate the specific biological functions of chromatin-remodeling enzymes with their biochemical behaviors. In model organisms such as budding yeast, it is relatively simple to align large groups of genes by their transcriptional start site. When this is done, a striking organization of nucleosomes is observed in which the region just upstream of the promoter is depleted for nucleosomes and an array of ordered nucleosomes extends into the coding region (Figure 4; Rando and Chang, 2009; Zhang et al., 2011). Superposed on the organization of nucleosomes, the distribution of many histone modifications, histone variants, and transcription-related factors show characteristic distributions across the averaged coding gene (Rando and Chang, 2009). Recently, it has emerged that a subset of chromatin-remodeling enzymes also exhibit distinct distributions with respect to transcribed genes (Figure 4; Yen et al., 2012). Below, we discuss how the reactions directed by different enzymes are incorporated into the broader context of transcription-coupled chromatin organization.

### Chromatin Organization

In budding yeast, the Chd1 and Isw1 proteins are found to be enriched in the coding regions of transcribed genes. Deletion of Chd1 alone results in a loss of regular spacing between nucleosomes within coding regions (Gkikopoulos et al., 2011). This is consistent both with previous studies linking Chd1 to the elongation of transcription (Simic et al., 2003) and with the biochemical properties of Chd1 that enable it to space arrays of nucleosomes on plasmid DNA (Lusser et al., 2005). The combined deletion of Chd1 and Isw1 results in a more profound loss of positioning (Gkikopoulos et al., 2011). A similar situation is observed in *S. pombe*, in which deletion of the two Chd1 homologs is required to disrupt nucleosome spacing (Hennig et al., 2012; Pointner et al., 2012; Shim et al., 2012). In both organisms, Chd1 proteins are functioning with partial redundancy. A consequence of this is that the defects in gene expression occurring upon deletion of Chd1 are restricted to the few genes in which Chd1 action is not redundant with another process.

It is attractive to speculate that the Chd1 and Isw1 proteins act to space nucleosomes as they are reassembled following transient dissociation during transcription. This reassembly reaction is likely to be rapid and assisted by histone chaperones such as FACT and Spt6. Remodeling enzymes such as ACF and Chd1 may assist this process, facilitating the conversion of chromatin assembly intermediates into nucleosomes (Torigoe et al., 2011). In *Drosophila*, Chd1 has been observed to interact with the histone chaperone HIRA and to participate in nonreplicative chromatin assembly (Konev et al., 2007).

In yeast, deletion of ISW1 and CHD1 correlates with increased histone exchange (Smolle et al., 2012). It is possible that the irregular spacing of nucleosomes in the absence of these enzymes renders them prone to dissociation as a result of collisions between adjacent nucleosomes (Engeholm et al., 2009). Transient dissociation of histones in the absence of Chd1 and Isw1 provides an opportunity for exchange with the soluble pool of nascent histones. Indeed, increased histone exchange is observed in the absence of Isw1 and Chd1, and this results in increased incorporation of acetylated histones over coding regions (Radman-Livaja et al., 2012; Smolle et al., 2012). As a result, in the absence of Chd1 and Isw1, nucleosomes over coding regions become hyperacetylated in addition to substantially losing positioning. Although changes in transcription of coding regions in this state are small, significant increases in noncoding transcription have been observed (Smolle et al., 2012). These observations illustrate how the action of ATP-dependent remodeling enzymes can be integrated with processes such as transcription and histone modification to sculpt the chromatin landscape.

Although deletion of Isw1 and Chd1 results in a loss of positioning of coding region nucleosomes, the nucleosome-free region and +1 nucleosome are largely unaffected (Gkikopoulos et al., 2011). This raises the possibility that another process is required to direct the positioning of the +1 nucleosome and that downstream nucleosomes are subsequently positioned with reference to this nucleosome. The Isw2 protein is known to influence the positioning of the +1 nucleosome (Whitehouse et al., 2007) and is localized to the +1 nucleosome by ChIP (Yen et al., 2012). Fine analysis of formaldehyde crosslinks using exonuclease digestion reveals extended contacts of Isw2 bound to +1 nucleosomes on the 5′ side of the gene (Yen et al., 2012). This provides evidence that linker DNA is bound by Isw2 on the 5′ side of nucleosomes that are repositioned in this direction in vivo. These results illustrate the power of genomic approaches to provide mechanistic insight. This study also found that binding of Reb1 was often observed adjacent to the region crosslinked to Isw2. Thus, Isw2 may bind +1 nucleosomes and reposition them adjacent to tightly bound transcription factors such as Reb1. The +1 nucleosome could then act as a reference point for the spacing of arrays of coding region nucleosomes directed by the Isw1 and Chd1 proteins (Zhang et al., 2011). Key questions that remain include how nucleosomes are organized immediately following DNA replication before they are transcribed and how nucleosomes are organized across the large regions of heterochromatin found in the genomes of higher eukaryotes.

### Chromatin Disruption

Depletion of RSC results in a partial filling in of the nucleosome-depleted region upstream of promoters (Hartley and Madhani, 2009), consistent with a role in nucleosome removal. RSC may be targeted to these regions through interactions with abundant transcription factors or its own DNA-binding specificity (Badis et al., 2008; Hartley and Madhani, 2009), and its action could provide one mechanism to reduce nucleosome occupancy at key regulatory elements. Similarly, in mammalian cells, human SWI/SNF complexes play roles in regulating chromatin organization at regulatory elements both pre- and postrecruitment of regulators (Burd and Archer, 2013).

The nature of the alteration to chromatin occurring at sites of SWI/SNF recruitment has not been characterized in all cases. However, examples exist to support nucleosome repositioning, disruption, and histone removal in different contexts. Recent studies provide evidence that these different activities of RSC may be functionally linked. RSC and SWI/SNF can move two nucleosomes into such close proximity that DNA is unwound from the histone octamer at the interface of the two nucleosomes (Dechassa et al., 2010; Engeholm et al., 2009; Ulyanova and Schnitzler, 2005). The loss of histone DNA contacts has been observed to result in dissociation of both histone dimers and, subsequently, histones H3 and H4. EM structures of RSC and SWI/SNF reveal a large binding cavity that can accommodate a nucleosome (Tang et al., 2010). The bound nucleosome appears to be used as a ram, destabilizing nucleosomes that it collides with (Dechassa et al., 2010). As a result, it would be expected that a single nucleosome would not be removed from DNA as effectively as one surrounded by neighbors. Consistent with this expectation, RSC removes nucleosomes more effectively from multinucleosome templates (Dechassa et al., 2010). During remodeling of the PHO5 regulatory region in vivo, a single nucleosome is retained (Boeger et al., 2008), and in addition, RSC bound to partially unwrapped nucleosomes has been detected at regulatory elements (Floer et al., 2010).

Interestingly, when native repressed PHO5 chromatin is incubated with RSC, the promoter nucleosomes are removed selectively, and this effect is sensitive to treatment of the template with a histone deacetylase (Lorch et al., 2011). This implicates histone acetylation as playing an important role in histone removal by RSC. This could occur via a simple tethering effect, as the RSC complex contains bromodomains that interact specifically with histones acetylated at H3 K14 (Kasten et al., 2004) and with the acetylated Rsc4 subunit (VanDemark et al., 2007). However, the acetylation of histones not only increases the binding of remodeling complexes, but also increases repositioning (Chatterjee et al., 2011) and dissociation of histones by RSC (Ferreira et al., 2007). At a structural level, the binding of RSC to histone tail peptides has been observed to result in a change in the conformation of the RSC complex (Skiniotis et al., 2007). Acetylation of histone H3 has been shown to increase the binding of specific regions of the H3 tail to the Snf2, Arp7, and Arp8 subunits of SWI/SNF (Chatterjee et al., 2011). This re-emphasizes the possibility raised in the earlier section that a change in the type of interaction between a remodeling enzyme and nucleosomes can alter the outcome of remodeling. Additional support for this stems from the finding that artificially tethering the chromotranslocase region of Chd1 to histones causes the enzyme to reposition nucleosomes in a fashion more similar to RSC or SWI/SNF than intact Chd1 (Patel et al., 2012). This illustrates the potential for histone contacts to influence the specificity of remodeling by both targeting and altering the outcome of remodeling reactions.

The genome-wide distributions of both RSC and SWI/SNF subunits indicate the presence of a tail of occupancy extending from promoters into the nucleosomes of the ORF (Yen et al., 2012). It’s possible that this reflects a function relating to the elongation of transcription, as both RSC (Soutourina et al., 2006) and SWI/SNF have been shown to have roles in elongation (Schwabish and Struhl, 2007). It is tempting to speculate that this role involves assisting the removal of histones from DNA during transcription by RNA polymerase.

### ATP-Dependent Histone Exchange

The prototypical remodeling enzyme linked to histone exchange is the Swr1 complex, which directs the replacement of nucleosomal histone H2A/H2B dimers with H2AZ/H2B variant dimers with high specificity (Mizuguchi et al., 2004). The yeast Swr1 complex is a 14 subunit complex, and both the Swc2 and Swr1 subunits directly interact with H2AZ (Wu et al., 2009). The ATPase activity of the Swr1 complex is activated by H2A-containing nucleosomes, but not H2AZ-containing nucleosomes (Luk et al., 2010). ATP hydrolysis is then further stimulated in the presence of free H2AZ/H2B dimers (Luk et al., 2010). The complex is capable of replacing the H2A/H2B dimers in a nucleosome in a stepwise reaction. This results in a nucleosome containing two H2AZ/H2B dimers, which is a nonoptimal substrate for the enzyme and thereby helps to provide directionality to the exchange process. It is likely that the conserved ATPase domains with Swr1 enzymes are tuned for the purpose of histone exchange. The spacing between conserved helicase-related motifs III and IV is larger in Swr1-related proteins compared to other Snf2-related proteins (Flaus et al., 2006). Within the Rad54 crystal structure (Thomä et al., 2005), the insertion site forms helical protrusions and a linker that are well placed to contact the substrate as it engages with the catalytic site. Within Swr1-related proteins, the insertions may serve to adapt DNA translocation at the catalytic site for the purpose of histone dimer exchange.

In *Saccharomyces cerevisiae*, the Ino80 and Fun30 proteins also have large insertions between motifs III and IV and share additional sequence homology with Swr1 proteins. Homologs of all three proteins have been identified in many of the sequenced genomes of eukaryae, indicating specialization for distinct functions. Both Ino80 and Fun30 have been shown to be capable of directing histone dimer exchange, but neither direct specific incorporation of H2AZ (Awad et al., 2010; Papamichos-Chronakis et al., 2011). Ino80 is most efficient in removing H2AZ/H2B and replacing it with H2A/H2B (Papamichos-Chronakis et al., 2011), whereas Fun30 exchanges H2AZ and H2A equally (Awad et al., 2010). Together, all three enzymes act to influence the distribution of H2AZ in vivo (Durand-Dubief et al., 2012; Mizuguchi et al., 2004; Papamichos-Chronakis et al., 2011). This illustrates there are at least three ways to influence the presence of a histone variant: targeted incorporation illustrated by Swr1, targeted removal as illustrated by Ino80, and increased exchange as illustrated by Fun30. It is possible that similar principles will apply to the distribution of other histone variants or modifications. Indeed, it has been proposed that posttranslational modification of H2AZ may act to regulate its distribution (Papamichos-Chronakis et al., 2011). The human ortholog of the Swr1 complex, TIP60, has combined chromatin remodeling and histone acetyltransferase activities (Doyon et al., 2004), and the human ortholog of Fun30 has profound effects on the re-establishment of histone modifications following DNA replication (Rowbotham et al., 2011).

The function of the histone variant H2AZ is most clearly defined at promoters where it flanks the nucleosome-free region and has been found to prevent the spread of heterochromatin (Raisner et al., 2005). However, the Swr1, Ino80, and Fun30 proteins are all also found in other regions of the genome and thus are likely to have additional functions (Durand-Dubief et al., 2012; Mizuguchi et al., 2004; Yen et al., 2012). A striking example is the finding that Fun30 influences the rate and extent of strand resection occurring during the repair of double-stranded DNA breaks (Chen et al., 2012; Costelloe et al., 2012; Eapen et al., 2012). Previous biochemical studies suggest that Fun30 is more likely to cause this effect by altering histone composition prior to resection. Consistent with this possibility, the action of Fun30 in strand resection is partially redundant with Ino80 and RSC (Chen et al., 2012).

Another mechanism for histone exchange is suggested by the case of ATRX. The human ATRX protein associates with the histone H3.3-specific chaperone DAXX to couple chromatin dissociation with the reassembly of nucleosomes enriched for this specific histone variant (Law et al., 2010; Lewis et al., 2010). The translocase domains of ATRX proteins have diverged from those of ISWI, Chd1, and Swr1 proteins. As a consequence, it is possible that they have not been adapted to engage with DNA on the surface of nucleosomes. This does not necessarily mean that ATRX proteins do not alter chromatin structure because this could still occur as a secondary consequence of DNA translocation initiating on linker DNA. In such a situation, one default outcome could be that of a snowplow, in which nucleosomes are nudged along DNA until they dissociate (Finkelstein et al., 2010). In the case of ATRX, coupling with DAXX could confer specificity for incorporation of H3.3. There are other examples illustrating the potential of the action of Snf2 proteins and histone chaperones to be combined (Lusser et al., 2005).

### Roles in Disease

Many chromatin-remodeling enzymes are conserved from yeast through to humans (Table 1), and to date, many of the functional paradigms established in yeast have relevance to a broad range of model organisms. In some cases, the composition of complexes has been found to be more complex in mammalian cells. For example, the subunit composition of the human SWI/SNF complexes purified from different cell lines varies (Kadoch et al., 2013). Whereas three distinct ISWI complexes have been identified in yeast, seven have been identified in humans (Erdel and Rippe, 2011). The functions of these complexes seem to have expanded to incorporate the increased complexity of mammalian cells. These include interactions with proteins only found in higher eukaryotes, such as steroid hormone receptors (Burd and Archer, 2013) and heterochromatin proteins (Ho et al., 2011), and involvement in processes such as differentiation and reprogramming (Singhal et al., 2010).

Mutations in components of human remodeling complexes have now been identified at high frequencies in human cancers (Table 2). Mutations in components of human SWI/SNF complexes are especially common and have been found to occur at a frequency of 19% across a spectrum of human cancers (Kadoch et al., 2013). This compares to a figure of 26% for p53, the most frequently mutated tumor suppressor. Mutations to other ATP-dependent remodeling enzymes including Chd1, Chd4, and ATRX and other chromatin-related factors are also detected in a range of cancers and in a range of albeit reduced frequency in comparison to SWI/SNF components (Garraway and Lander, 2013). One possible explanation underlying the association of SWI-SNF mutations with cancer is that the complex contributes to genome stability. Consistent with this, anaphase bridges are observed at high frequency following inactivation of BRG1 (Dykhuizen et al., 2013). However, the specificity with which inactivation of different subunits affects different types of cancer (Table 2) suggests more complex tissue specific modes of action (Kadoch et al., 2013).

### Conclusions

The function of only a limited subset of the 24 subfamilies of Snf2-related proteins has been discussed above. Nonetheless, these cases illustrate some of the principles via which an ancient DNA-translocating core is subject to fine regulation that adapts it for a diverse range of functions. These functions often fit within pathways that intersect with genetic processes such as transcription, DNA replication, and DNA repair and with other forms of chromatin alteration such as posttranslational modification of histones that act to shape the chromatin landscape on a genome scale. In some cases, it is emerging that remodeling enzymes have roles in human disease that are more widespread than the links to relatively rare syndromes characterized previously. In many cases, alterations to the function of Snf2-related proteins appear to be selected for at high frequency in tumor development. This provides renewed motivation to take advantage of the battery of new experimental approaches suited to providing new insight into the structure, mechanism, and functions of this diverse family of proteins.

## Figures and Tables

**Table 1:** Subfamilies of Snf2-Related Proteins

	Human	*Drosophila*	*Arabidopsis*	*Cervisiae*
1	SMARCA4 (BRG1) and SMARCA2 (BRM)	Brahma	CHR2 (ATBRM), CHR3 (SYD), CHR23, and CHR12	Snf2/Swi2 and Sth1
2	hSNF2H(SMARCA5) and hSNF2L(SMARCA1)	ISWI	CHR11 and CH17,	Isw2 and Isw1
3	CHD1 and CHD2	CHD1	CHR5	Chd1
4	CHD3, CHD3, CHD4, and CHD5	Mi-2 and Chd3	CHR6 (CKH2, EPP1, PICKLE, and SSL2), CHR4 (PKR1), and CHR7 (PKR2)	
5	CHD6, CHD7, CHD8, and CHD9	KISMET		
6	HELLS (LSH, PASG, and SMARCA6)		DDM1 (CHR1, SOM1, and CHA1)	IRC5
7	ALC1 (CHD1L)		ASG3 (CHR10)	

8	SRCAP (DOMO1 and SWR1)	Domino	CHR13 (PIE1 and SRCAP)	Swr1
9	EP400			
10	INO80	Ino80	INO80	Ino80
11	ETL1 (SMARCAD1)	Etl1	CHR19 (ETL1)	Fun30

12	RAD54L and RAD54B	okra (rad54)	CHR25 (RAD54)	Rad54 and Rdh54
13	ATRX	XNP (dATRX)	CHR20 (ATRX)	
14	RAD54L2	CG4049		
15			CHR38 (CLSY), CHR42, CHR34, CHR35 (DRD1 and DMS1), and CHR40.	

16	HLTF (SMARCA3 and SNF2L3)		RAD5, AT5G43530, ATG05120, ATG5130, and AT1G02670	Rad5 and Rad16
17			AT1G50410, AT3G20010, AT1G61140 (EDA16), AT1G11100, and AT3G16600	Ris1
18	TTF2	Lodestar (horka) and CG10445		
19	SHPRH	CG7376	AT2G40770 and AT3G54460	IRC20
20	BTAF1 (TAF172)	Hel89B (BTAF1)	BTAF1 (CHR16 and RGD3)	Mot1
21	ERCC6 (CSB and RAD26), ERCC6L (PICH and RAD26L), and ERCC6L2		CHR8, CHR9, and CHR24	Rad26

22	SMARCAL1 (HARP) and ZRANB3	Marcal1	CHR18 and AT5G07810	

Names of Snf2-related proteins in different species, with synonyms in brackets. Related subfamilies are grouped based upon sequence homology within the translocase domains (Flaus et al., 2006). The proteins in rows 1 to 7 are most closely related to Snf2 and often participate in reactions that involve nucleosome repositioning. The Swr1-related proteins grouped in rows 8–11 share an activity in exchange of histone dimers. The distinguishing mechanistic characteristics of other subfamilies are not yet known.

**Table 2:** Links between Remodeling Enzymes and Cancer

Tumor	Frequency Mutation Observed	Genes Mutated^a^
Renal clear cell carcinoma	41%	PBRM subunit of human SWI/SNF BAF complex
Ovarian clear cell carcinoma	75%	ARID1A (BAF250a), ARID1B (BAF250b), SMARCA4 (BRG1), and BCL11A subunits of human SWI/SNF BAF complex
Colorectal cancer	55%	Many subunits of human SWI/SNF complexes
Pancreatic cancer	10%	Predominantly BRG1 and ARID1A subunits of human SWI/SNF complexes
Melanoma	39%	Many subunits of human SWI/SNF complexes
Synovial sarcoma	95%	SS18 subunit of human SWI/SNF BAF complex fused to SSX proteins
Malignant Rhabdoid tumors	∼100%	BAF47 (hSNF5)
Hepatocellular carcinoma	33%	Predominantly ARID1A, ARID1B, and ARID2 subunits of human SWI/SNF complexes
Lung cancer	35%	All subunits of human SWI/SNF complexes mutated
Breast cancer	11%	Many subunits of human SWI/SNF complexes mutated
Pancreatic neuroendocrine tumours, glioblastoma	45%	ATRX, DAAX, and histone H3.3
Prostate cancer	∼20%	Chd1
Endometrial cancer	17%	Chd4

a See Garraway and Lander (2013) and Kadoch et al. (2013).

## Supplemental Information

Table S1. Cues Influencing the Regulation of Snf2-Related ATPases

## References

[bib1] Awad S., Ryan D., Prochasson P., Owen-Hughes T., Hassan A.H. (2010). The Snf2 homolog Fun30 acts as a homodimeric ATP-dependent chromatin-remodeling enzyme. J. Biol. Chem..

[bib2] Badis G., Chan E.T., van Bakel H., Pena-Castillo L., Tillo D., Tsui K., Carlson C.D., Gossett A.J., Hasinoff M.J., Warren C.L. (2008). A library of yeast transcription factor motifs reveals a widespread function for Rsc3 in targeting nucleosome exclusion at promoters. Mol. Cell.

[bib3] Bétous R., Mason A.C., Rambo R.P., Bansbach C.E., Badu-Nkansah A., Sirbu B.M., Eichman B.F., Cortez D. (2012). SMARCAL1 catalyzes fork regression and Holliday junction migration to maintain genome stability during DNA replication. Genes Dev..

[bib4] Blosser T.R., Yang J.G., Stone M.D., Narlikar G.J., Zhuang X.W. (2009). Dynamics of nucleosome remodelling by individual ACF complexes. Nature.

[bib5] Blossey R., Schiessel H. (2008). Kinetic proofreading of gene activation by chromatin remodeling. HFSP J.

[bib6] Boeger H., Griesenbeck J., Kornberg R.D. (2008). Nucleosome retention and the stochastic nature of promoter chromatin remodeling for transcription. Cell.

[bib7] Böhm V., Hieb A.R., Andrews A.J., Gansen A., Rocker A., Tóth K., Luger K., Langowski J. (2011). Nucleosome accessibility governed by the dimer/tetramer interface. Nucleic Acids Res..

[bib8] Burd C.J., Archer T.K. (2013). Chromatin architecture defines the glucocorticoid response. Mol. Cell. Endocrinol..

[bib9] Chatterjee N., Sinha D., Lemma-Dechassa M., Tan S., Shogren-Knaak M.A., Bartholomew B. (2011). Histone H3 tail acetylation modulates ATP-dependent remodeling through multiple mechanisms. Nucleic Acids Res..

[bib10] Chen X., Cui D., Papusha A., Zhang X., Chu C.D., Tang J., Chen K., Pan X., Ira G. (2012). The Fun30 nucleosome remodeller promotes resection of DNA double-strand break ends. Nature.

[bib11] Clapier C.R., Cairns B.R. (2009). The biology of chromatin remodeling complexes. Annu. Rev. Biochem..

[bib12] Clapier C.R., Cairns B.R. (2012). Regulation of ISWI involves inhibitory modules antagonized by nucleosomal epitopes. Nature.

[bib13] Costelloe T., Louge R., Tomimatsu N., Mukherjee B., Martini E., Khadaroo B., Dubois K., Wiegant W.W., Thierry A., Burma S. (2012). The yeast Fun30 and human SMARCAD1 chromatin remodellers promote DNA end resection. Nature.

[bib14] Côté J., Quinn J., Workman J.L., Peterson C.L. (1994). Stimulation of GAL4 derivative binding to nucleosomal DNA by the yeast SWI/SNF complex. Science.

[bib15] Dang W.W., Bartholomew B. (2007). Domain architecture of the catalytic subunit in the ISW2-nucleosome complex. Mol. Cell. Biol..

[bib16] Dechassa M.L., Sabri A., Pondugula S., Kassabov S.R., Chatterjee N., Kladde M.P., Bartholomew B. (2010). SWI/SNF has intrinsic nucleosome disassembly activity that is dependent on adjacent nucleosomes. Mol. Cell.

[bib17] Deindl S., Hwang W.L., Hota S.K., Blosser T.R., Prasad P., Bartholomew B., Zhuang X. (2013). ISWI remodelers slide nucleosomes with coordinated multi-base-pair entry steps and single-base-pair exit steps. Cell.

[bib18] Doyon Y., Selleck W., Lane W.S., Tan S., Côté J. (2004). Structural and functional conservation of the NuA4 histone acetyltransferase complex from yeast to humans. Mol. Cell. Biol..

[bib19] Durand-Dubief M., Will W.R., Petrini E., Theodorou D., Harris R.R., Crawford M.R., Paszkiewicz K., Krueger F., Correra R.M., Vetter A.T. (2012). SWI/SNF-like chromatin remodeling factor Fun30 supports point centromere function in S. cerevisiae. PLoS Genet..

[bib20] Dürr H., Körner C., Müller M., Hickmann V., Hopfner K.P. (2005). X-ray structures of the Sulfolobus solfataricus SWI2/SNF2 ATPase core and its complex with DNA. Cell.

[bib21] Dykhuizen E.C., Hargreaves D.C., Miller E.L., Cui K., Korshunov A., Kool M., Pfister S., Cho Y.-J., Zhao K., Crabtree G.R. (2013). BAF complexes facilitate decatenation of DNA by topoisomerase IIα. Nature.

[bib22] Eapen V.V., Sugawara N., Tsabar M., Wu W.H., Haber J.E. (2012). The Saccharomyces cerevisiae chromatin remodeler Fun30 regulates DNA end resection and checkpoint deactivation. Mol. Cell. Biol..

[bib23] Eberharter A., Ferrari S., Längst G., Straub T., Imhof A., Varga-Weisz P., Wilm M., Becker P.B. (2001). Acf1, the largest subunit of CHRAC, regulates ISWI-induced nucleosome remodelling. EMBO J..

[bib24] Engeholm M., de Jager M., Flaus A., Brenk R., van Noort J., Owen-Hughes T. (2009). Nucleosomes can invade DNA territories occupied by their neighbors. Nat. Struct. Mol. Biol..

[bib25] Erdel F., Rippe K. (2011). Chromatin remodelling in mammalian cells by ISWI-type complexes—where, when and why?. FEBS J..

[bib26] Fairman-Williams M.E., Guenther U.P., Jankowsky E. (2010). SF1 and SF2 helicases: family matters. Curr. Opin. Struct. Biol..

[bib27] Ferreira H., Flaus A., Owen-Hughes T. (2007). Histone modifications influence the action of Snf2 family remodelling enzymes by different mechanisms. J. Mol. Biol..

[bib28] Finkelstein I.J., Visnapuu M.L., Greene E.C. (2010). Single-molecule imaging reveals mechanisms of protein disruption by a DNA translocase. Nature.

[bib29] Flaus A., Martin D.M.A., Barton G.J., Owen-Hughes T. (2006). Identification of multiple distinct Snf2 subfamilies with conserved structural motifs. Nucleic Acids Res..

[bib30] Floer M., Wang X., Prabhu V., Berrozpe G., Narayan S., Spagna D., Alvarez D., Kendall J., Krasnitz A., Stepansky A. (2010). A RSC/nucleosome complex determines chromatin architecture and facilitates activator binding. Cell.

[bib31] Fyodorov D.V., Blower M.D., Karpen G.H., Kadonaga J.T. (2004). Acf1 confers unique activities to ACF/CHRAC and promotes the formation rather than disruption of chromatin in vivo. Genes Dev..

[bib32] Garraway L.A., Lander E.S. (2013). Lessons from the cancer genome. Cell.

[bib33] Gkikopoulos T., Schofield P., Singh V., Pinskaya M., Mellor J., Smolle M., Workman J.L., Barton G.J., Owen-Hughes T. (2011). A role for Snf2-related nucleosome-spacing enzymes in genome-wide nucleosome organization. Science.

[bib34] Gorbalenya A.E., Koonin E.V. (1993). Helicases: amino acid sequence comparisons and structure-function relationships. Curr. Opin. Struct. Biol..

[bib35] Grüne T., Brzeski J., Eberharter A., Clapier C.R., Corona D.F., Becker P.B., Müller C.W. (2003). Crystal structure and functional analysis of a nucleosome recognition module of the remodeling factor ISWI. Mol. Cell.

[bib36] Hartley P.D., Madhani H.D. (2009). Mechanisms that specify promoter nucleosome location and identity. Cell.

[bib37] Hauk G., Bowman G.D. (2011). Structural insights into regulation and action of SWI2/SNF2 ATPases. Curr. Opin. Struct. Biol..

[bib38] Hauk G., McKnight J.N., Nodelman I.M., Bowman G.D. (2010). The chromodomains of the Chd1 chromatin remodeler regulate DNA access to the ATPase motor. Mol. Cell.

[bib39] He X., Fan H.Y., Narlikar G.J., Kingston R.E. (2006). Human ACF1 alters the remodeling strategy of SNF2h. J. Biol. Chem..

[bib40] Hennig B.P., Bendrin K., Zhou Y., Fischer T. (2012). Chd1 chromatin remodelers maintain nucleosome organization and repress cryptic transcription. EMBO Rep..

[bib41] Ho L., Miller E.L., Ronan J.L., Ho W.Q., Jothi R., Crabtree G.R. (2011). esBAF facilitates pluripotency by conditioning the genome for LIF/STAT3 signalling and by regulating polycomb function. Nat. Cell Biol..

[bib42] Hota S.K., Bhardwaj S.K., Deindl S., Lin Y.C., Zhuang X., Bartholomew B. (2013). Nucleosome mobilization by ISW2 requires the concerted action of the ATPase and SLIDE domains. Nat. Struct. Mol. Biol..

[bib43] Kadoch C., Hargreaves D.C., Hodges C., Elias L., Ho L., Ranish J., Crabtree G.R. (2013). Proteomic and bioinformatic analysis of mammalian SWI/SNF complexes identifies extensive roles in human malignancy. Nat. Genet..

[bib44] Kagalwala M.N., Glaus B.J., Dang W.W., Zofall M., Bartholomew B. (2004). Topography of the ISW2-nucleosome complex: insights into nucleosome spacing and chromatin remodeling. EMBO J..

[bib45] Kasten M., Szerlong H., Erdjument-Bromage H., Tempst P., Werner M., Cairns B.R. (2004). Tandem bromodomains in the chromatin remodeler RSC recognize acetylated histone H3 Lys14. EMBO J..

[bib46] Konev A.Y., Tribus M., Park S.Y., Podhraski V., Lim C.Y., Emelyanov A.V., Vershilova E., Pirrotta V., Kadonaga J.T., Lusser A., Fyodorov D.V. (2007). CHD1 motor protein is required for deposition of histone variant H3.3 into chromatin in vivo. Science.

[bib47] Law M.J., Lower K.M., Voon H.P.J., Hughes J.R., Garrick D., Viprakasit V., Mitson M., De Gobbi M., Marra M., Morris A. (2010). ATR-X syndrome protein targets tandem repeats and influences allele-specific expression in a size-dependent manner. Cell.

[bib48] Lewis R., Dürr H., Hopfner K.P., Michaelis J. (2008). Conformational changes of a Swi2/Snf2 ATPase during its mechano-chemical cycle. Nucleic Acids Res..

[bib49] Lewis P.W., Elsaesser S.J., Noh K.M., Stadler S.C., Allis C.D. (2010). Daxx is an H3.3-specific histone chaperone and cooperates with ATRX in replication-independent chromatin assembly at telomeres. Proc. Natl. Acad. Sci. USA.

[bib50] Lia G., Praly E., Ferreira H., Stockdale C., Tse-Dinh Y.C., Dunlap D., Croquette V., Bensimon D., Owen-Hughes T. (2006). Direct observation of DNA distortion by the RSC complex. Mol. Cell.

[bib51] Lorch Y., Griesenbeck J., Boeger H., Maier-Davis B., Kornberg R.D. (2011). Selective removal of promoter nucleosomes by the RSC chromatin-remodeling complex. Nat. Struct. Mol. Biol..

[bib52] Luk E., Ranjan A., Fitzgerald P.C., Mizuguchi G., Huang Y., Wei D., Wu C. (2010). Stepwise histone replacement by SWR1 requires dual activation with histone H2A.Z and canonical nucleosome. Cell.

[bib53] Lusser A., Urwin D.L., Kadonaga J.T. (2005). Distinct activities of CHD1 and ACF in ATP-dependent chromatin assembly. Nat. Struct. Mol. Biol..

[bib54] McKnight J.N., Jenkins K.R., Nodelman I.M., Escobar T., Bowman G.D. (2011). Extranucleosomal DNA binding directs nucleosome sliding by Chd1. Mol. Cell. Biol..

[bib55] Mizuguchi G., Shen X., Landry J., Wu W.H., Sen S., Wu C. (2004). ATP-driven exchange of histone H2AZ variant catalyzed by SWR1 chromatin remodeling complex. Science.

[bib56] Morra R., Lee B.M., Shaw H., Tuma R., Mancini E.J. (2012). Concerted action of the PHD, chromo and motor domains regulates the human chromatin remodelling ATPase CHD4. FEBS Lett..

[bib57] Mueller-Planitz F., Klinker H., Ludwigsen J., Becker P.B. (2013). The ATPase domain of ISWI is an autonomous nucleosome remodeling machine. Nat. Struct. Mol. Biol..

[bib58] Narlikar G.J. (2010). A proposal for kinetic proof reading by ISWI family chromatin remodeling motors. Curr. Opin. Chem. Biol..

[bib59] Papamichos-Chronakis M., Watanabe S., Rando O.J., Peterson C.L. (2011). Global regulation of H2A.Z localization by the INO80 chromatin-remodeling enzyme is essential for genome integrity. Cell.

[bib60] Patel A., Chakravarthy S., Morrone S., Nodelman I.M., McKnight J.N., Bowman G.D. (2012). Decoupling nucleosome recognition from DNA binding dramatically alters the properties of the Chd1 chromatin remodeler. Nucleic Acids Res..

[bib61] Pointner J., Persson J., Prasad P., Norman-Axelsson U., Strålfors A., Khorosjutina O., Krietenstein N., Svensson J.P., Ekwall K., Korber P. (2012). CHD1 remodelers regulate nucleosome spacing in vitro and align nucleosomal arrays over gene coding regions in S. pombe. EMBO J..

[bib62] Racki L.R., Yang J.G., Naber N., Partensky P.D., Acevedo A., Purcell T.J., Cooke R., Cheng Y.F., Narlikar G.J. (2009). The chromatin remodeller ACF acts as a dimeric motor to space nucleosomes. Nature.

[bib63] Radman-Livaja M., Quan T.K., Valenzuela L., Armstrong J.A., van Welsem T., Kim T., Lee L.J., Buratowski S., van Leeuwen F., Rando O.J., Hartzog G.A. (2012). A key role for Chd1 in histone H3 dynamics at the 3′ ends of long genes in yeast. PLoS Genet..

[bib64] Raisner R.M., Hartley P.D., Meneghini M.D., Bao M.Z., Liu C.L., Schreiber S.L., Rando O.J., Madhani H.D. (2005). Histone variant H2A.Z marks the 5′ ends of both active and inactive genes in euchromatin. Cell.

[bib65] Rando O.J., Chang H.Y. (2009). Genome-wide views of chromatin structure. Annu. Rev. Biochem..

[bib66] Rowbotham S.P., Barki L., Neves-Costa A., Santos F., Dean W., Hawkes N., Choudhary P., Will W.R., Webster J., Oxley D. (2011). Maintenance of silent chromatin through replication requires SWI/SNF-like chromatin remodeler SMARCAD1. Mol. Cell.

[bib67] Ryan D.P., Sundaramoorthy R., Martin D., Singh V., Owen-Hughes T. (2011). The DNA-binding domain of the Chd1 chromatin-remodelling enzyme contains SANT and SLIDE domains. EMBO J..

[bib68] Saha A., Wittmeyer J., Cairns B.R. (2002). Chromatin remodeling by RSC involves ATP-dependent DNA translocation. Genes Dev..

[bib69] Saha A., Wittmeyer J., Cairns B.R. (2005). Chromatin remodeling through directional DNA translocation from an internal nucleosomal site. Nat. Struct. Mol. Biol..

[bib70] Schwabish M.A., Struhl K. (2007). The Swi/Snf complex is important for histone eviction during transcriptional activation and RNA polymerase II elongation in vivo. Mol. Cell. Biol..

[bib71] Schwanbeck R., Xiao H., Wu C. (2004). Spatial contacts and nucleosome step movements induced by the NURF chromatin remodeling complex. J. Biol. Chem..

[bib72] Sharma A., Jenkins K.R., Héroux A., Bowman G.D. (2011). Crystal structure of the chromodomain helicase DNA-binding protein 1 (Chd1) DNA-binding domain in complex with DNA. J. Biol. Chem..

[bib73] Shim Y.S., Choi Y., Kang K., Cho K., Oh S., Lee J., Grewal S.I., Lee D. (2012). Hrp3 controls nucleosome positioning to suppress non-coding transcription in eu- and heterochromatin. EMBO J..

[bib74] Simic R., Lindstrom D.L., Tran H.G., Roinick K.L., Costa P.J., Johnson A.D., Hartzog G.A., Arndt K.M. (2003). Chromatin remodeling protein Chd1 interacts with transcription elongation factors and localizes to transcribed genes. EMBO J..

[bib75] Singhal N., Graumann J., Wu G., Araúzo-Bravo M.J., Han D.W., Greber B., Gentile L., Mann M., Schöler H.R. (2010). Chromatin-Remodeling Components of the BAF Complex Facilitate Reprogramming. Cell.

[bib76] Singleton M.R., Dillingham M.S., Wigley D.B. (2007). Structure and mechanism of helicases and nucleic acid translocases. Annu. Rev. Biochem..

[bib77] Sirinakis G., Clapier C.R., Gao Y., Viswanathan R., Cairns B.R., Zhang Y. (2011). The RSC chromatin remodelling ATPase translocates DNA with high force and small step size. EMBO J..

[bib78] Skiniotis G., Moazed D., Walz T. (2007). Acetylated histone tail peptides induce structural rearrangements in the RSC chromatin remodeling complex. J. Biol. Chem..

[bib79] Smolle M., Venkatesh S., Gogol M.M., Li H., Zhang Y., Florens L., Washburn M.P., Workman J.L. (2012). Chromatin remodelers Isw1 and Chd1 maintain chromatin structure during transcription by preventing histone exchange. Nat. Struct. Mol. Biol..

[bib80] Soutourina J., Bordas-Le Floch V., Gendrel G., Flores A., Ducrot C., Dumay-Odelot H., Soularue P., Navarro F., Cairns B.R., Lefebvre O., Werner M. (2006). Rsc4 connects the chromatin remodeler RSC to RNA polymerases. Mol. Cell. Biol..

[bib81] Stockdale C., Flaus A., Ferreira H., Owen-Hughes T. (2006). Analysis of nucleosome repositioning by yeast ISWI and Chd1 chromatin remodeling complexes. J. Biol. Chem..

[bib82] Tan S., Davey C.A. (2011). Nucleosome structural studies. Curr. Opin. Struct. Biol..

[bib83] Tang L., Nogales E., Ciferri C. (2010). Structure and function of SWI/SNF chromatin remodeling complexes and mechanistic implications for transcription. Prog. Biophys. Mol. Biol..

[bib84] Thomä N.H., Czyzewski B.K., Alexeev A.A., Mazin A.V., Kowalczykowski S.C., Pavletich N.P. (2005). Structure of the SWI2/SNF2 chromatin-remodeling domain of eukaryotic Rad54. Nat. Struct. Mol. Biol..

[bib85] Torigoe S.E., Urwin D.L., Ishii H., Smith D.E., Kadonaga J.T. (2011). Identification of a rapidly formed nonnucleosomal histone-DNA intermediate that is converted into chromatin by ACF. Mol. Cell.

[bib86] Ulyanova N.P., Schnitzler G.R. (2005). Human SWI/SNF generates abundant, structurally altered dinucleosomes on polynucleosomal templates. Mol. Cell. Biol..

[bib87] VanDemark A.P., Kasten M.M., Ferris E., Heroux A., Hill C.P., Cairns B.R. (2007). Autoregulation of the rsc4 tandem bromodomain by gcn5 acetylation. Mol. Cell.

[bib88] Watson A.A., Mahajan P., Mertens H.D., Deery M.J., Zhang W., Pham P., Du X., Bartke T., Zhang W., Edlich C. (2012). The PHD and chromo domains regulate the ATPase activity of the human chromatin remodeler CHD4. J. Mol. Biol..

[bib89] Whitehouse I., Stockdale C., Flaus A., Szczelkun M.D., Owen-Hughes T. (2003). Evidence for DNA translocation by the ISWI chromatin-remodeling enzyme. Mol. Cell. Biol..

[bib90] Whitehouse I., Rando O.J., Delrow J., Tsukiyama T. (2007). Chromatin remodelling at promoters suppresses antisense transcription. Nature.

[bib91] Wu W.H., Wu C.H., Ladurner A., Mizuguchi G., Wei D., Xiao H., Luk E., Ranjan A., Wu C. (2009). N terminus of Swr1 binds to histone H2AZ and provides a platform for subunit assembly in the chromatin remodeling complex. J. Biol. Chem..

[bib92] Yamada K., Frouws T.D., Angst B., Fitzgerald D.J., DeLuca C., Schimmele K., Sargent D.F., Richmond T.J. (2011). Structure and mechanism of the chromatin remodelling factor ISW1a. Nature.

[bib93] Yang J.G., Madrid T.S., Sevastopoulos E., Narlikar G.J. (2006). The chromatin-remodeling enzyme ACF is an ATP-dependent DNA length sensor that regulates nucleosome spacing. Nat. Struct. Mol. Biol..

[bib94] Yen K., Vinayachandran V., Batta K., Koerber R.T., Pugh B.F. (2012). Genome-wide nucleosome specificity and directionality of chromatin remodelers. Cell.

[bib95] Zhang Y., Smith C.L., Saha A., Grill S.W., Mihardja S., Smith S.B., Cairns B.R., Peterson C.L., Bustamante C. (2006). DNA translocation and loop formation mechanism of chromatin remodeling by SWI/SNF and RSC. Mol. Cell.

[bib96] Zhang Z., Wippo C.J., Wal M., Ward E., Korber P., Pugh B.F. (2011). A packing mechanism for nucleosome organization reconstituted across a eukaryotic genome. Science.

[bib97] Zofall M., Persinger J., Kassabov S.R., Bartholomew B. (2006). Chromatin remodeling by ISW2 and SWI/SNF requires DNA translocation inside the nucleosome. Nat. Struct. Mol. Biol..

