# Supplementary material for: Mechanisms and Functions of ATP-Dependent Chromatin-Remodeling Enzymes
Source: Cell. 2013 Aug 1;154(3):490–503. doi: 10.1016/j.cell.2013.07.011 (PMC3781322; doi:10.1016/j.cell.2013.07.011)
Supplement: Table S1. Cues Influencing the Regulation of Snf2-Related ATPases [file mmc1.pdf]

Table S1. Cues Influencing the Regulation of Snf2-Related ATPases

Substrate	Example	References
Transcriptional activation domains	The Swi1 and Snf5 subunits of SWI/SNF interact with acidic transcriptional activation domains. Similarly, steroid hormone receptors with human SWI/SNF.	(Burd and Archer, 2013; Prochasson et al., 2003; Yudkovsky et al., 1999)
Basal transcription factors	Mot1 ATPases include HEAT repeats directing specificity to TBP.	(Wollmann et al., 2011)
RNA Polymerase elongation factors	Chd1 interacts with the Rtf1 subunit of the Paf1 elongation complex.	(Simic et al., 2003)
Transcriptional repressors	The yeast Isw2 complex interacts with the repressor Ume6.	(Goldmark et al., 2000)
Histones	The activity of ISWI enzymes is regulated by an epitope in the histone H4 tail.	(Hamiche et al., 2001)
Post-translational modifications	In many cases folds with the potential to recognise histone modifications are present e.g. Bromo, Chromo, PHD, CUE, Macro domains.	(Clapier and Cairns, 2009)
Histone variants	The Swc2 and Swr1 subunits of the Swr1 complex interact specifically with H2AZ-H2B histone dimers.	(Wu et al., 2009)
Histone chaperones	The association of the ATRX complex with the histone chaperone DAAX confers specificity for H3.3.	(Law et al., 2010; Lewis et al., 2010)
DNA	The RSC3 subunit of the yeast RSC complex shows specificity in its binding to DNA. Non specific DNA binding domains within other remodeling enzymes may direct them to DNA adjacent to nucleosomes.	(Badis et al., 2008)
DNA structure	The HARP2 domains of SMARCAL1 target this enzyme to branched DNA structures.	(Betous et al., 2012)

Supplemental References

Badis, G., Chan, E.T., van Bakel, H., Pena-Castillo, L., Tillo, D., Tsui, K., Carlson, C.D., Gossett, A.J., Hasinoff, M.J., Warren, C.L.*, et al.* (2008). A Library of Yeast Transcription Factor Motifs Reveals a Widespread Function for Rsc3 in Targeting Nucleosome Exclusion at Promoters. Molecular Cell *32*, 878-887.

Betous, R., Mason, A.C., Rambo, R.P., Bansbach, C.E., Badu-Nkansah, A., Sirbu, B.M., Eichman, B.F., and Cortez, D. (2012). SMARCAL1 catalyzes fork regression and Holliday junction migration to maintain genome stability during DNA replication. Genes & Development *26*, 151-162.

Burd, C.J., and Archer, T.K. (2013). Chromatin architecture defines the glucocorticoid response. Mol Cell Endocrinol.

Clapier, C.R., and Cairns, B.R. (2009). The Biology of Chromatin Remodeling Complexes. Annual Review of Biochemistry *78*, 273-304.

Goldmark, J.P., Fazzio, T.G., Estep, P.W., Church, G.M., and Tsukiyama, T. (2000). The Isw2 chromatin remodeling complex represses early meiotic genes upon recruitment by Ume6p. Cell *103*, 423-433.

Hamiche, A., Kang, J.G., Dennis, C., Xiao, H., and Wu, C. (2001). Histone tails modulate nucleosome mobility and regulate ATP-dependent nucleosome sliding by NURF. Proc Natl Acad Sci U S A *98*, 14316-14321.

Law, M.J., Lower, K.M., Voon, H.P.J., Hughes, J.R., Garrick, D., Viprakasit, V., Mitson, M., De Gobbi, M., Marra, M., Morris, A.*, et al.* (2010). ATR-X Syndrome Protein Targets Tandem Repeats and Influences Allele-Specific Expression in a Size-Dependent Manner. Cell *143*, 367-378.

Lewis, P.W., Elsaesser, S.J., Noh, K.M., Stadler, S.C., and Allis, C.D. (2010). Daxx is an H3.3-specific histone chaperone and cooperates with ATRX in replication-independent chromatin assembly at telomeres. Proceedings of the National Academy of Sciences of the United States of America *107*, 14075-14080.

Prochasson, P., Neely, K.E., Hassan, A.H., Li, B., and Workman, J.L. (2003). Targeting activity is required for SWI/SNF function in vivo and is accomplished through two partially redundant activator-interaction domains. Mol Cell *12*, 983-990.

Simic, R., Lindstrom, D.L., Tran, H.G., Roinick, K.L., Costa, P.J., Johnson, A.D., Hartzog, G.A., and Arndt, K.M. (2003). Chromatin remodeling protein Chd1 interacts with transcription elongation factors and localizes to transcribed genes. EMBO Journal *22*, 1846-1856.

Wollmann, P., Cui, S., Viswanathan, R., Berninghausen, O., Wells, M.N., Moldt, M., Witte, G., Butryn, A., Wendler, P., Beckmann, R.*, et al.* (2011). Structure and mechanism of the Swi2/Snf2 remodeller Mot1 in complex with its substrate TBP. Nature *475*, 403-407.

Wu, W.H., Wu, C.H., Ladurner, A., Mizuguchi, G., Wei, D., Xiao, H., Luk, E., Ranjan, A., and Wu, C. (2009). N terminus of Swr1 binds to histone H2AZ and provides a platform for subunit assembly in the chromatin remodeling complex. J Biol Chem *284*, 6200-6207.

Yudkovsky, N., Logie, C., Hahn, S., and Peterson, C.L. (1999). Recruitment of the SWI/SNF chromatin remodeling complex by transcriptional activators. Genes & Development *13*, 2369-2374.
